# Mixed Signals and Interspecies Variation in the Plasticity of Adult Mammal Brains

**DOI:** 10.3390/cells15060520

**Published:** 2026-03-13

**Authors:** Alessia Pattaro, Marco Ghibaudi, Alessandro Zanone, Valentina Cerrato, Chet C. Sherwood, Luca Bonfanti

**Affiliations:** 1 Neuroscience Institute Cavalieri Ottolenghi, 10043 Orbassano, Italy; alessia.pattaro@unito.it (A.P.); marco.ghibaudi@unito.it (M.G.); alessandro.zanone@edu.unito.it (A.Z.); valentina.cerrato@unito.it (V.C.); 2 Department of Veterinary Sciences, University of Turin, 10095 Grugliasco, Italy; 3 Department of Neuroscience Rita Levi-Montalcini, University of Turin, 10126 Turin, Italy; 4 Department of Anthropology and Center for the Advanced Study of Human Paleobiology, The George Washington University, Washington, DC 20052, USA; sherwood@gwu.edu

**Keywords:** brain plasticity, arrested maturation, adult neurogenesis, doublecortin, comparative neuroplasticity, neuron, mammals, primates

## Abstract

**Bullet points:**

- Increased understanding of brain structural plasticity in mammals has led to increasing conceptual complexity and conflicting results in the field.
- The growing recognition of interspecies variation in neuroplasticity, from mice to humans, and the overlapping of cell markers in different cell populations are the main elements of confusion.
- Some cell types, cell markers, and biological processes markedly differ among species due to the non-linear sculpting that took place during evolution, including trade-offs in plasticity.
- The discovery of non-dividing, immature (late-maturing) neurons has increased the heterogeneity in brain plasticity and can explain some controversial findings.
- Understanding how brain structural plasticity has adapted to diverse mammalian neuroanatomies is essential for meaningful translational research.

**Abstract:**

Despite the growing interest in brain structural plasticity and the substantial body of knowledge that has accumulated over recent decades, some issues remain poorly defined, leading to confusion in the interpretation of results. In addition to stem cell-driven neurogenesis in adult neurogenic niches (adult neurogenesis), neuronal precursors in a state of arrested maturation have also been described, representing a form of neurogenesis without division based on so-called “immature” or late-maturing neurons. These processes occur in different brain regions yet share certain molecular markers and temporal windows. Recent advances in comparative neuroplasticity have further complicated our understanding. Studies reveal a reduction in adult neurogenesis in the olfactory bulb and hippocampus of large-brained, gyrencephalic mammals compared with small-brained species such as rodents. Conversely, a higher prevalence of immature neurons has been reported in the neocortex and amygdala of larger-brained mammals. It is becoming evident that evolutionary trade-offs took place in distinct plastic processes, resulting in the predominance of certain forms in particular species, while others coexist and share overlapping markers. Regardless of the approach employed (neuroanatomical, immunocytochemical, phylogenetic, or transcriptional), current evidence indicates substantial heterogeneity in cell types with different origins and fates across diverse mammalian species. These patterns appear to be sculpted by evolutionary pressures yet unified by shared transient maturational states.

## 1. Introduction

Structural plasticity is a property of the brain’s nervous tissue and allows physical changes to occur in the fine anatomical organization of neural circuits over time. Such changes may play a role in multiple aspects of brain function at different stages of the lifespan, such as (i) the sculpting of neural circuit organization and connections during early life periods, when the brain is still growing [1,2,3]; (ii) the maintenance of flexibility in the adult brain to adapt to a changing environment [2,4]; (iii) the maintenance of brain function efficiency during aging (so-called cognitive reserve or brain reserve [5]); and (iv) brain repair after nerve tissue lesion (trauma, ischemia, or stroke) or in the course of neurological disorders [6]. The possibility of the latter is reduced in mammals due to evolutionary constraints [7,8,9]. Another hypothesis is that brain plasticity may have a particularly important preventive role in humans across their lives in reducing the impact or delaying the onset of dementia by maintaining cognitive reserve [10,11,12].

At the cellular level, brain structural changes include the formation and elimination of synaptic contacts (synaptic plasticity), various forms of glial and neural–glial cell plasticity (including changes in white matter bundles and connectome), or the creation of new neurons and their integration into preexisting circuits (canonical neurogenesis [3,13,14,15,16,17,18,19,20]). The latter process has captivated the scientific community over the last three decades, as it is linked to the persistence of active neural stem cells, increasing the optimism for achieving brain repair through regenerative medicine [6,21]. Nevertheless, most brain plasticity, including stem cell-driven neurogenic processes, is not limited to a reparative role: it also plays a role in “protracted developmental programs”, allowing for the refinement of specific brain circuits during youth, during which individuals’ brains are sculpted based on experience through exploring the world [1,2,4,9,22]. Accordingly, structural plasticity types and rates are age-related and substantially vary across animal species and brain regions and are related to their different lifespans.

Recent comparative studies—involving a wide range of species beyond the typical rodent models (including humans) as well as different approaches (histology, immunocytochemistry, connectomics, and transcriptomics) applied to different contexts, spanning from transgenic animal models to postmortem analysis of very large brains—have revealed substantial variation in brain structural plasticity across species [9,23,24,25]. Overall, a picture is emerging in which evolutionary pressures linked to different ecological niches may have sculpted a different mix of plasticity types in each species, involving various cell populations in different brain regions [9,26]. To explain such heterogeneity, which is characterized by nuances rather than sharp distinctions, the concept of non-canonical neurogenesis has been introduced [27] (see below).

**Figure 1:**
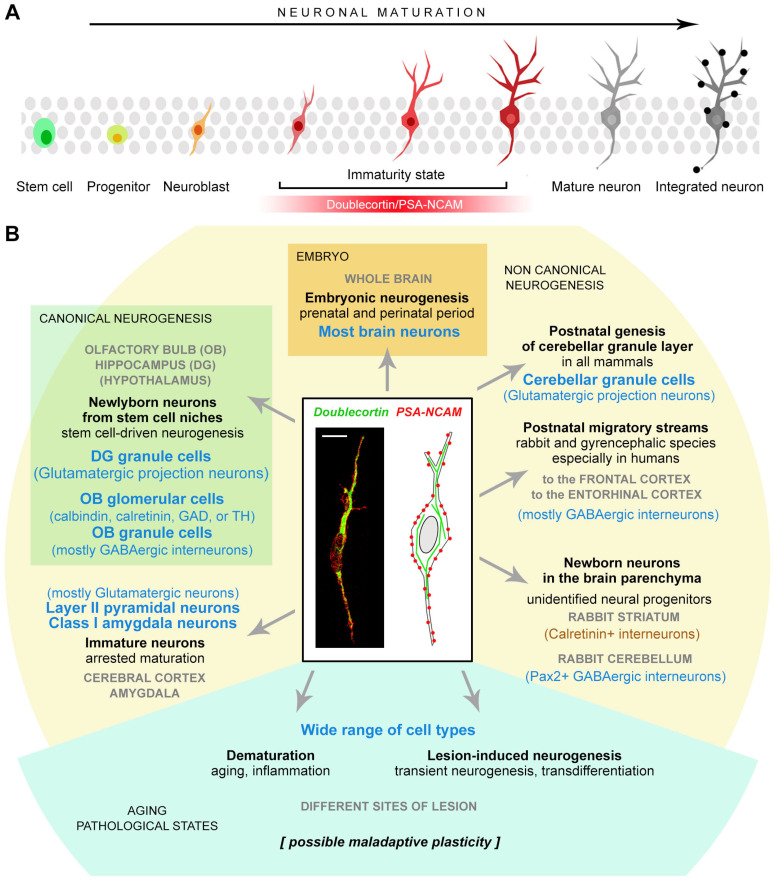
Heterogeneity of cell types representing the outcome of different plastic processes and anatomical locations in mammalian brains, with cells passing through undifferentiated states that share the same markers. (**A**) Neuronal maturation is a multistep process from stem cell division to the integration of a functional neuron into a circuit. The phase of immaturity (in red), characterized by the transient expression of cytoskeletal (DCX) and membrane-bound (PSA-NCAM) markers, can be markedly different in length depending on cell type, brain region, and animal species. (**B**) Widely different neuronal cell types (blue characters) can originate from the DCX^+^/PSA-NCAM^+^ immature cells at different anatomical locations (capital, gray text) through different plastic processes (bold black text) that can differ among mammal species. Scale bar: 10 µm.

Despite cell type heterogeneity and interspecies differences, most cell populations involved in such plasticity changes share a phase of immaturity in which they appear very similar and express the same markers (Figure 1), e.g., the cytoskeletal protein doublecortin (DCX [28]) and the polysialylated form of the neural cell adhesion molecule (PSA-NCAM [29]). These commonalities in expression profiles have led to confusion over the years [30,31]. This review aims to clarify some principles of mammalian brain structural plasticity to better define major gaps in current knowledge and to highlight new avenues for future research in the field.

## 2. Non-Canonical Neurogenic Processes

**Figure 2:**
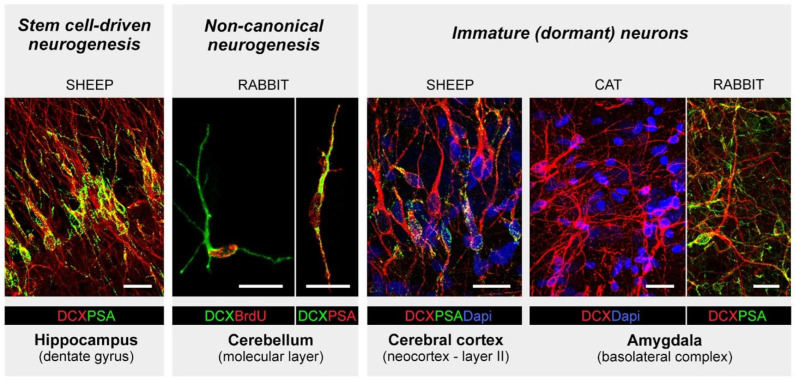
Very similar young neuronal elements, spanning from neuroblast-like cells to undifferentiated neurons, all expressing the common markers of immaturity, doublecortin (DCX) and polysialylated N-CAM (PSA-NCAM), are detectable in the young and adult brain, intermixed with fully mature neurons. These immature cells can be found in widely different contexts, spanning different biological (plastic) processes and different brain regions. Some examples are given, including stem cell-driven neurogenesis within canonical neurogenic sites, parenchymal neurogenesis from widespread neural progenitors in the cerebellum, and immature neurons in the cerebral cortex and amygdala. Such heterogeneity is present qualitatively and quantitatively when considering different mammal species. Images were reproduced from Refs. [52,53,54]; all images were distributed under the terms of the Creative Commons Attribution License, which permits unrestricted use and redistribution provided that the original author and source are credited. Scale bars: 20 µm.

This term was originally created [27] to consider different neurogenic processes occurring in the brain parenchyma outside the “canonical” neurogenic sites characterized by active neural stem cells hosted in morphologically well-defined niches. Such neurogenic niches are present in the wall of the lateral ventricle (subventricular zone, SVZ) and in the hippocampal dentate gyrus (subgranular zone, SGZ), where complete neurogenic processes may take place, varying from the division of bona fide stem cells to the functional integration of new neurons [32]. Canonical adult neurogenesis in the olfactory bulb and hippocampus has been studied intensively over the last 30 years, unveiling the cell types, cell markers and molecular mechanisms involved in this process in a few animal models (mostly laboratory rodents [18,19,33,34]). Non-canonical neurogenesis was identified more recently when comparative studies started to reveal remarkable variation across mammals [23,26,35,36,37]. This has sometimes generated confusion, with most researchers exclusively using (and referring to) laboratory rodents (reviewed and discussed in [30,38,39,40,41,42]) and then finding discrepancies with data obtained in humans [30,43,44,45,46,47,48,49,50]. The landscape of comparative brain structural plasticity research is further complicated by two facts: (i) the non-canonical addition of new neurons can occur through different means, and (ii) some markers commonly used to identify immature, highly plastic or migrating cells and undifferentiated neurons (DCX and PSA-NCAM) are actually shared by different cell types in different brain regions, as well as at different developmental stages of cells (summarized in Figure 1 and Figure 2; see [31,51] and below). In what follows, the main examples of such heterogeneity, along with a discussion of some sources of confusion, are presented.

### 2.1. Neurogenesis Outside Canonical Neurogenic Niches: Parenchymal Neurogenesis

In addition to stem cell-driven adult neurogenesis, some processes may initiate in neural progenitors located outside of the stem cell niches, referred to as parenchymal neurogenesis [55,56,57]. This can happen either through division of stem-like cells not arriving at a clearly identifiable, functional outcome (e.g., the tanycytes located in the third ventricular wall of the hypothalamus [58,59,60]) or through the genesis of neuronal elements within restricted temporal windows, as described in the rabbit cerebellum [52,61] and in the guinea pig and rabbit striatum [56,62]. A similar example is the production of new glial cells in the absence of a clearly defined stem cell niche, such as the oligodendrocyte progenitors in both white and gray matter parenchyma [63].

**Figure 3:**
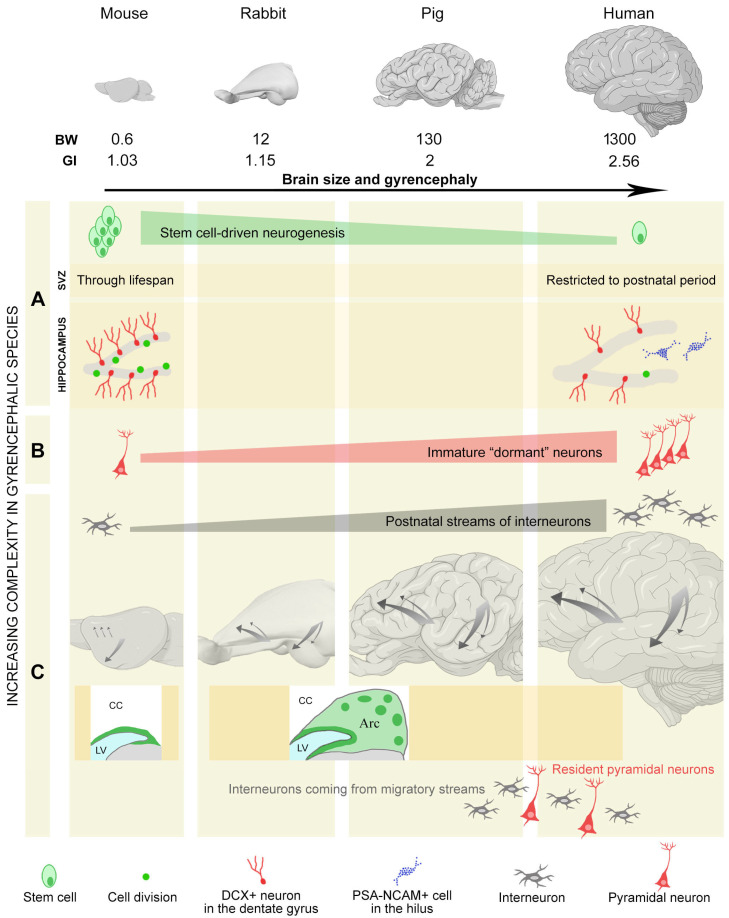
Heterogeneity of brain plasticity in diverse mammals endowed with different brain sizes and gyrification. BW, brain weight (in grams); GI, gyrification index. (**A**,**B**) Quantitative difference between stem cell-driven neurogenesis and immature or dormant neurons. (**A**) Quantitative and qualitative differences in canonical neurogenic sites between rodents and humans: general reduction in humans (**top**), widely different periods of protracted neurogenesis in the subventricular zone (SVZ), and differences in the hippocampus with high rates of cell division (green dots) in rodents that are scarce or absent in humans, in which PSA-NCAM-positive (DCX-negative) multipolar cells are present in the hilus (blue [71]). (**B**) Remarkable increase in immature neurons from small-brained to large-brained species (in cerebral cortex and amygdala). (**C**), Increase in postnatal addition of interneurons in the cortex of gyrencephalic species, particularly humans, through streams of DCX^+^ neuroblasts (gray arrows). Note that, in rabbits, this process is supported by an expanded subventricular zone containing large chains of neuroblasts (Arc) that is not present in rodents. LV, lateral ventricle; CC, corpus callosum. The interplay between (**B**) and (**C**) increases the complexity of structural plasticity in the cortex, where incoming interneurons are mixed with resident immature neurons. Brain icons created with BioRender.com.

In the caudate nucleus of young adult rabbits, clusters of proliferating cells located within the parenchyma give rise to chains of DCX^+^/PSA-NCAM^+^ neuroblasts, some of which ultimately differentiate into newly formed neurons (calretinin-positive striatal interneurons [56]). A variant of the parenchymal neurogenic process described in rabbits has also been found in guinea pigs, where the striatal genesis of neuronal precursors is transient, occurring during juvenile life and peaking at weaning [62]. The parenchymal genesis of neuronal elements might be the product of vestigial stem-like progenitor cells dispersed during embryonic neurogenesis or during a protracted (perinatal/postnatal) migration of neuronal precursors. Indeed, rabbits are also characterized by multiple chain-like organized neuroblasts and an expanded SVZ (the so-called Arc), recently described in gyrencephalic species (see Section 2.2 and Section 5.3 and Figure 3C). Though striatal parenchymal neurogenesis is not detectable in rodents, a very similar, transient process can be induced in the mouse striatum after experimental excitotoxic lesion or stroke, producing DCX^+^/PSA-NCAM^+^ chains of neuroblasts originating from activated astrocytes and differentiating into transient, still unidentified neuronal elements [64,65,66]. These findings introduce a further level of complexity in non-canonical neurogenesis, consisting of transdifferentiation of glial (stem-like) elements into neurogenic astrocytes that are not part of the canonical stem cell niches [67].

Rabbits have also been shown to display a peculiar process of neurogenesis in the cerebellum, a region that, in all mammals, is characterized by the postnatal genesis of granule cells through persistence of a mitotically active layer (external granule cell layer, EGL; located in the subpial position), which remains non-neurogenic throughout life [68,69,70]. In rabbits, the EGL is replaced by a proliferative layer called the subpial layer (SPL [61]), which persists beyond puberty, forming chains of DCX^+^/PSA-NCAM^+^ neuroblasts on the cerebellar surface. In parallel, but independently from the SPL, a neurogenic process giving rise to cells expressing the transcription factor Pax2, a marker for GABAergic cerebellar interneuron precursors, occurs in the molecular layer [52]. These are progenitors of neuroepithelial origin that ascend through the white matter from the wall of the fourth ventricle. The neurogenic process continues to a lesser extent in adulthood, after exhaustion of the proliferative SPL [52] (Figure 2).

All these cases involve the production of undifferentiated neuronal cells expressing markers of immaturity within the mature brain parenchyma, though in different ways. Their outcomes can be widely different, though not yet completely characterized [27] (Figure 1 and Figure 2).

In parallel, putative neurogenic processes occurring at several brain parenchymal locations have been proposed over the years, including the cerebral cortex of macaque monkeys [72,73] and rats [74] and the amygdala of monkeys [75,76]. Nevertheless, the findings reported in these studies were not subsequently confirmed, likely due to confusion arising from the shared cellular markers of immaturity discussed here, as well as the alternative explanation provided by the dormant neuron hypothesis (see Section 2.3 and Figure 1).

Overall, while canonical neurogenic processes can be considered a well-conserved feature in the olfactory bulb and hippocampus of most mammals (apart from differences in quantitative and age-related variation), non-canonical, parenchymal neurogenesis appears to be a highly species-specific exception.

### 2.2. Postnatal Migration of Young Neurons to Their Committed Destination

In addition to stem cell-driven and parenchymal neurogenesis, other forms of plasticity that provide new neurons in preexisting circuits have been described [55,77,78,79,80,81,82,83], for example, perinatal/postnatal migration of young neuronal populations to some cortical areas at very early life stages. This has been especially observed in large-brained gyrencephalic species, including humans (reviewed in [81,84,85]; Figure 3C). These cells include young GABAergic interneurons, which reach the anterior cingulate gyrus in the human frontal cortex during the first three months of life [77] and the entorhinal cortex within the temporal lobe during the first three years [79]. Most of these cortical inhibitory neurons originate from delayed neurogenesis in the caudal ganglionic eminence; their number and overall proportion are greater in primates than in rodents [85,86,87]. Accordingly, the occurrence of chains of DCX^+^/PSA-NCAM^+^ cells was recently confirmed in the gyrencephalic brain of piglets, with streams directed to the medial prefrontal cortex, the frontal cortex, the cingulate cortex, and the piriform and entorhinal cortices [82,83]. Supplementary data published in Kim et al. [82] also indicate the occurrence of similar chains in the brains of sheep and chimpanzees.

The postnatal addition of interneurons in species with larger brains may support the evolution of more complex cognitive functions, and it has recently been considered as a feature linked to gyrencephaly [82]. Nevertheless, the first examples of multiple chains of neuroblasts directed to the cortex were observed in the brain of rabbits, namely, non-gyrencephalic mammals [55,61]. Elaborated SVZ-derived migratory streams exist in young rabbits (3–6 months old), both anteriorly, towards the frontal cortex [55,61], and posteriorly, through chains of neuroblasts in a ventral–lateral extension of the SVZ adjacent to the external capsule and directed towards the piriform and perirhinal cortices [55]. An average of 5–6 chains anteriorly and 40–50 chains posteriorly are detectable, which is remarkably different compared to observations in rodents and marmosets, where these structures are barely detectable [55,61,82]. The elaborate subventricular zone, characterized by large masses of neuroblasts and supporting the postnatal migration of cortical interneurons, has been recently termed the Arc [82]. The Arc has been observed in the brains of humans and piglets [80,82,83], as well as in rabbits [55,61], but is absent in lissencephalic marmosets and mice [82].

Of importance, the migration of prenatally generated neuronal precursors to their postnatal committed destination is an early life process mainly observed to occur in large-brained, gyrencephalic species, but not rodents. In mice, most neuronal precursors generated in the postnatal SVZ converge into the rostral migratory stream to reach the olfactory bulb [19]. Only a small, secondary migratory pathway has been found to reach the basal forebrain [88], and sparse cells have been observed to reach the cortex through the dorsal corpus callosum white matter [89,90]. Comparative studies suggest that the above-mentioned migratory routes are a conserved developmental process, mostly occurring prenatally and perinatally in rodents, while they are more protracted in time in larger, gyrencephalic brains.

In addition to these well-defined migratory routes, it has been suggested that slow migration of neuronal precursors might also continue to occur for extended periods of time, possibly reaching the amygdala and claustrum [85]. Shorter-range migration of excitatory immature neurons might also occur during adolescence close to the amygdala paralaminar nucleus and basolateral complex of different mammals, including humans [91]. Nevertheless, a full demonstration of such a process is currently lacking, and the morphology and directional aspects of the immature cells in the amygdala of some gyrencephalic species might contradict such a hypothesis [53]. On this basis, several past studies have suggested the occurrence of protracted/persistent neurogenic processes in these regions (see, for example, [75,76,92]). More recent reports have revealed that these regions host populations of immature, non-dividing neurons [53,91,93]. Hence, due to the above-mentioned postnatal streams, especially in non-rodent species at juvenile ages, a mix of two populations of young neurons can coexist, namely incoming interneurons and resident, immature pyramidal neurons (Figure 3C), further increasing the complexity of structural plasticity in certain species with large brains.

### 2.3. Immature or Dormant Neurons

The concept of non-canonical neurogenesis has recently been extended with the identification of prenatally generated neuronal precursors that can retain an undifferentiated state for long periods of time (“immature” or “dormant” neurons) [31,91,94,95,96,97,98,99,100,101]. These late-maturing, postmitotic cells were first described in the paleocortex of rodents (piriform cortex [102,103]), then revealed as prenatally generated, postmitotic elements by injection in the embryo of 5′-bromo-2′-deoxyuridine (BrdU [94]). More recently, using DCX-Cre-ERT2/Flox-EGFP transgenic mice, in which immature DCX-expressing cells were labeled permanently in vivo at different life stages, it was demonstrated that the cortical immature neurons can progressively “awaken” to mature into glutamatergic pyramidal neurons [98]. The full maturation of these neuronal precursors is supported by increased dendritic ramification and dendritic spine density, by the appearance of the axon initial segment, and by their functional integration in the cortical circuitry, evident by the generation of electric activity [98,99]. The dormant precursors are capable of awakening at all ages but are far more abundant during youth [104,105]; thus, their awakening slows down at older ages [105]. Little is known regarding the cellular and molecular mechanisms behind their state of arrested maturation, apart from the role of PSA-NCAM in maintaining their protracted state of immaturity [106]. We know that they can differentiate into pyramidal, excitatory neurons corresponding to the principal neurons of cortical layer II and the basolateral amygdala [53,91,98,99,107,108], but we lack information on their physiological role in the cortical and amygdalar circuits (see [108]).

**Figure 4:**
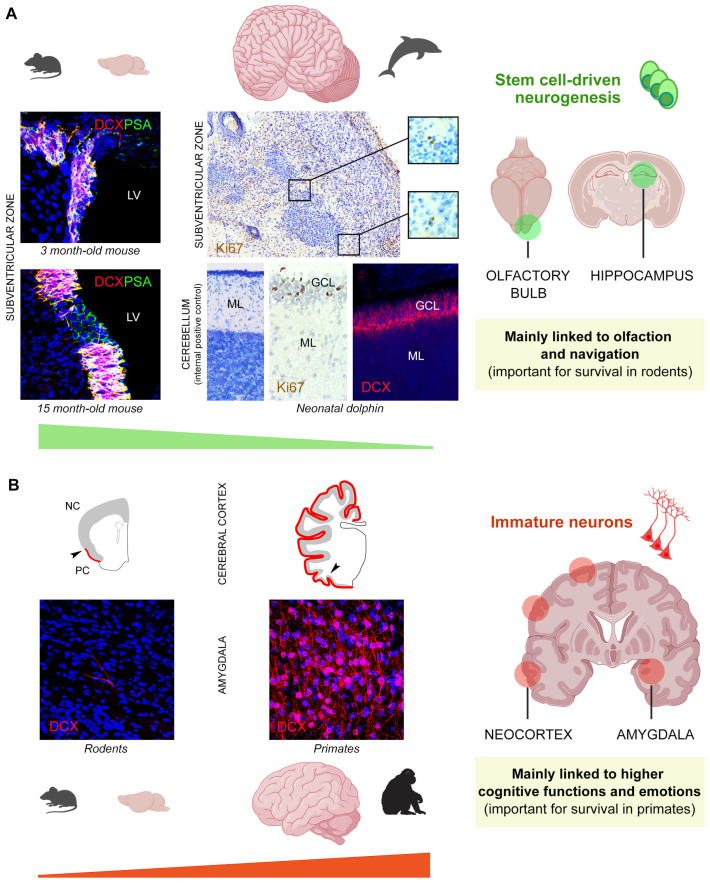
Extreme examples of interspecies differences concerning both canonical and non-canonical neurogenic processes in mammals adapted to widely different ecological niches. (**A**) Stem cell-driven neurogenesis (green) linked to olfaction dramatically decreases from small-brained rodents to large-brained dolphins. A very active periventricular neurogenic zone (subventricular zone, SVZ) persists throughout life in mice, while a vestigial SVZ appears substantially exhausted soon after birth in dolphins, which are large-brained cetaceans devoid of olfactory brain structures (modified with permission from [114]). Only a few dividing cells (Ki67-positive nuclei) are detectable in the dolphin SVZ-like region, mostly appearing in “doublets”, as described for oligodendrocyte progenitor cells. Bottom middle, internal positive control for DCX and Ki67 antigen immunoreactivity in the actively dividing granule cell layer (GCL) of the cerebellum of the same animals (modified with permission from [114]). DCX, doublecortin; PSA, polysialylated form of the neural cell adhesion molecule; LV, lateral ventricle; ML, molecular layer. (**B**) The opposite situation is detectable in the neocortex and amygdala of small-brained, lissencephalic species (rodents) and large-brained, gyrencephalic species (primates, sheep, horses, and dogs) concerning the occurrence of non-dividing, immature neurons (red), which are scarce in the former and extremely abundant in the latter. Red line (top), anatomical extension of the immature neurons in the cortical layer II. PC, paleocortex; NC, neocortex, separated by the arrowhead. Modified with permission from Ref. [109] (top) and [53] (bottom), distributed under the terms of the Creative Commons Attribution License, which permits unrestricted use and redistribution provided that the original author and source are credited. Brain icons created with BioRender.com.

Of importance, recent studies have revealed a high degree of heterogeneity of the immature neurons across animal species and brain regions: in large-brained, gyrencephalic mammals, they were found to extend within layer II of the whole cerebral cortex, including the neocortex [109,110], and to be extremely abundant both in the cortex and in the basolateral amygdala [53,91,93,111,112] (see Figure 3B and Figure 4B and Section 3 for more detail on phylogenetic variation).

Initially, due to their shared immaturity markers with the newborn neurons produced by adult neurogenesis, these DCX^+^/PSA-NCAM^+^ cells were incorrectly considered as a parenchymal form of neurogenesis, both in the cortex and amygdala [74,76,92]. Later studies involving BrdU injections followed by different survival times showed that the DCX^+^ cells of cortical layer II are generated during embryogenesis [94,95,107] and persist in the adult brain in a prolonged state of immaturity [94]. The occurrence of dividing oligodendrocyte progenitors, which are largely widespread in the brain parenchyma [63,113], can cause confusion, requiring studies of co-expression (or to exclude co-expression) with markers of neuronal immaturity [53,91].

## 3. Interspecies Differences in Mammalian Neuroplasticity

Comparative studies on neuroplasticity have intensified during recent decades. New data reveal significant interspecies differences concerning the rates and types of plasticity, their topographical location, the expression of cell markers (including species specificity of antibodies for immunocytochemistry), the time courses of neuronal maturation processes, and the transcriptional profiles of cell populations.

### 3.1. Stem Cell-Dependent and -Independent Neurogenesis: The Trade-Off Hypothesis

The identification of a population of “immature” or “dormant” neurons in the cerebral cortex [98,99,101], and more recently in the amygdala [53,91,93], has led to the definition of a new category of non-canonical, parenchymal neurogenesis [27,97,100,115,116]. This can be considered a form of “neurogenesis without division” [8] since it does not require the existence of stem cells and their niches; however, in the mouse piriform cortex, it has been shown to yield neurons capable of full maturation and functional integration into cortical circuits ([98,99,105]; see above). This alternative form of brain plasticity contributes to our increasing understanding of the heterogeneity of mechanisms underlying neurogenic events [101,109].

The immature cells in cortical layer II and the amygdala appear to be present in all mammal species studied so far [95,102,103,111,112,117,118,119,120,121,122,123,124] and share multiple features, indicating that they belong to the same cell population [53,91,93,98,99,107,109,110] (see Section 5). However, systematic quantification of the DCX^+^ immature neurons in the cerebral cortex [109,110] and amygdala [53] of eight phylogenetically diverse mammal species, including mice and chimpanzees, among others, revealed marked interspecies variation: while small numbers of immature neurons were found in rodents, a very large reserve of these cells was observed in large-brained, gyrencephalic species (Figure 3B and Figure 4B), with one order of magnitude difference in cell densities (number of DCX^+^ cells/mm or/mm^2^ of cortical perimeter or amygdala area, respectively) and several orders of magnitude difference in the estimated total number of immature cells per whole hemisphere or whole amygdala (dozens in rodents and millions in sheep, dogs, chimpanzees and horses considering the neocortex [53,109,110]).

Overall, in large-brained, gyrencephalic species, including humans and other primates, a mix of stem cell-dependent and stem cell-independent neurogenesis likely maintains reservoirs of undifferentiated neurons, as well as adds different types of new functional neurons at different time points and in different brain regions: (i) early addition of newborn neurons in the hippocampus and olfactory bulb; (ii) a contribution of prenatally generated migratory interneurons in the entorhinal and prefrontal cortex; and (iii) late maintenance of immature, dormant neurons in the whole cerebral cortex and amygdala. It is suggested that there was a trade-off of such a mix of plastic processes during evolution: stem cell-driven processes are less evident and less protracted in large-brained, gyrencephalic species capable of more elaborated, high-order cognitive functions and complex social interactions due to their widely expanded and more interconnected cortical mantles, while far greater numbers of immature neurons are hosted in cortical and subcortical regions [9,54,125]. A trade-off is an evolutionary concept referring to situations in which a compromise occurs between two or more traits that offer distinct benefits but cannot be fully optimized concurrently (due to anatomical limitations or to limited resources among competing demands; References in [9]). In the brains of mammals, despite all species possessing all types of plasticity, the rate and regional location of some plastic processes appear to have been differently implemented (or partially lost) by each animal species based on the ecological niche and evolutionary pressures that have modified its neuroanatomy (reviewed in [9,125]; see also [126] and Section 4). As pointed out recently [127], many exceptions exist, especially when considering animal species other than mammals, and other trade-off hypotheses have been proposed based on different aspects of embryonic neurogenesis, involving newly acquired genes linked to neocortex expansion and modifications in brain structure.

### 3.2. Cell Markers Across Species: Useful Tools or Confounding Signals

Cell markers represent a fundamental tool for studying neurodevelopmental biology, including the characterization of stem cells and their progeny [28,29,128,129,130,131]. Nevertheless, they can be misleading for several reasons, especially when dealing with highly dynamic processes such as neuronal maturation: (i) they can overlap in different biological processes (see below); (ii) they can be shared by different cell types; (iii) they can occur during different developmental time windows across species [132]; (iv) they can persist for a longer duration during the maturational process of different cell types [31]; and (v) they can be the result of dysregulated states, such as inflammation, excitotoxicity, or senescence [133].

An obvious yet often neglected point is that immaturity markers do not define cell types but rather a shared maturational state due to a transient molecular profile that can be found in widely different cell populations and that can be lost at different time points in the cell life (Figure 1). DCX and PSA-NCAM are two classic examples of this. In the past, they were associated with specific cellular processes (e.g., cell migration and neurogenesis [28,29]), but they can be expressed in a variety of plastic processes not involving cell genesis or migration (reviewed in [51]) and even in pathological or maladaptive states [134,135]. Furthermore, species-specific features exist in marker expression: for instance, in the adult human hippocampus, besides a reduction in cell genesis with respect to rodents (see Section 5.4), a population of PSA-NCAM-positive but DCX-negative neurons is present in the hilus, which is absent in rodents [44,71] (Figure 3A). For these reasons, immunocytochemical approaches have been recently implemented in parallel with transcriptomic analyses.

### 3.3. Cell Heterogeneity Across Species: Insights and Pitfalls from Single-Cell Transcriptomics

The advent of single-cell and single-nucleus transcriptomic (sc/snRNA-seq) technologies represents a major advance in the identification and description of neuroblasts and undifferentiated neurons across species [49,50]. These approaches allow cell states to be defined based on their whole transcriptional profile rather than on single markers traditionally used in the field, such as DCX or PSA-NCAM. Indeed, these sequencing technologies have clearly shown that DCX alone is an inadequate marker of neuronal immaturity. While DCX is strongly enriched in neuroblasts of the adult mouse dentate gyrus, its expression in pigs, macaques and humans is weaker and broadly distributed across other cell types, including inhibitory interneurons and glial cells [136]. Accordingly, in the neonatal human Arc, DCX^+^ cells have been shown to encompass heterogeneous GABAergic populations spanning multiple differentiation stages, from proliferative intermediate progenitors to postmitotic migratory neurons, indicating that DCX expression defines a broad window of neuronal immaturity rather than a discrete cell identity [82].

The power of sc/snRNA-seq lies in its ability to investigate neurogenic niches across species, to identify conserved and divergent features, and to uncover novel candidate markers. However, these approaches must be applied with particular care, as inappropriate analytical strategies can lead to false-negative or false-positive results and to data misinterpretation. Indeed, the application of sc/snRNA-seq to adult primate brains has yielded contradictory results regarding the existence and abundance of immature neurons [137]. Of importance, strategies that search for cells expressing markers identified in rodents that implicitly assume cross-species equivalence have led to false-negative results, as exemplified by Franjic et al. [136]. Conversely, approaches relying on the expression of a small number of genes—such as *DCX*, *PROX1* or *SNAP25* [138], or *LPAR1* [139]—may result in false-positive interpretations, since these few genes may also be expressed by other cell types/states [25,140]. Similarly, unsupervised cluster-based methods are often poorly suited to identifying extremely rare populations, such as immature neurons in adult human and nonhuman primate brains, and have, in some cases, generated misleading results. For example, Hao et al. [141] reported disproportionately large clusters of putative immature granule cells in the macaque hippocampus that were later shown to lack key immature neuronal features [25]. Earlier studies also misidentified ependymal cells as neural stem cells [47,142] or radial glia-like populations, which were later reinterpreted as astrocytic states [49,143], underscoring the difficulty of distinguishing progenitors from astroglial populations using clustering alone.

A promising alternative through machine learning-based approaches has been proposed [24,25], which identifies immature neuron-like cells at adult stages by leveraging transcriptional features from young individuals within the same species, rather than by cross-species comparison. This strategy enabled the detection of putative immature granule cells in both the macaque and human hippocampus and resolved inconsistencies across previously published datasets [136,138,141,143]. Importantly, rather than directly comparing gene expression across species—an approach that may be confounded by batch effects arising from differences in tissue processing, sequencing platforms, and sequencing depth—the authors compared immature and mature granule cells within each dataset. This strategy revealed highly divergent gene expression patterns across species that nevertheless converged onto shared biological processes [25]. However, concerns regarding reproducibility remain, as transcriptional signatures were derived from single datasets, precluding direct validation across independent studies within the same species. Moreover, this approach makes the underlying assumption that immature neuron-like cells detected at adult stages correspond to newly generated neurons and engage transcriptional programs that largely recapitulate those active during developmental neurogenesis. If long-lived “dormant” immature neurons also exist in the hippocampus, their molecular programs may only partially overlap with developmental neurogenesis, potentially limiting the ability of developmental reference-based approaches to detect or correctly classify them.

Despite these limitations, single-cell transcriptomics has uncovered candidate shared genes and species-specific genes associated with immature neurons, including conserved genes such as *DPYSL5*, *FGFR1*, *FNBP1L* and *NRP2*; primate-enriched genes such as *COL25A1*, *DSCAML1* and *SEMA3A* [25]; the primate-specific NSC marker *ETNPPL*; and immature GC markers *STMN1* and *STMN2* [24,143]. Notably, *STMN1* was later found to exhibit only a non-significant trend toward enrichment in immature GCs when more stringent analyses were performed [25], illustrating how marker identity is highly sensitive to cell definition and statistical thresholds. Moreover, many of these genes display enrichment rather than strict specificity for immature neurons and are also expressed, albeit at lower levels, in mature cells. Age-dependent transcriptional changes further complicate interpretation, as shown by the distinct age-related expression patterns of genes such as *NEUROD4* and *NFIA* in human, but not mouse, immature granule cells [24].

Together, these observations argue against defining immature neurons based on single markers and instead emphasize the need for multi-gene signatures or coordinated transcriptional programs that more robustly capture the continuum of neuronal maturation. They also highlight the need for systematic analyses across multiple datasets that sample the same populations within the same species to establish a consensus set of gene modules.

A major limitation of snRNA-seq remains the lack of spatial information. Recent spatial transcriptomics studies of the human hippocampus [50,144,145] have begun to address this issue by revealing spatially resolved, age-associated gene expression changes linked to neurogenesis and granule cell maturation. However, the limited resolution of current whole-transcriptome spatial platforms necessitates complementary approaches, such as single-molecule FISH, to validate candidate genes at the cellular resolution. Emerging high-resolution spatial technologies, particularly targeted approaches with near-single-cell resolution [146], will be instrumental in integrating transcriptional signatures with anatomical context and defining the spatial and temporal organization of neurogenic niches in adult brains. In the near future, the same analyses should be performed on the novel cell populations of putative “dormant” neurons, with special reference to large-brained, gyrencephalic species and humans. A systematic comparison of the immature signatures in different species and brain regions, including cortical layer II and the amygdala (to be compared with the hippocampus), is needed to better characterize the nature of the protracted maturation, while carefully accounting for the methodological limitations and analytical pitfalls discussed above.

## 4. Comparative Neuroplasticity: A Compromise Between Structural Changes and Functional Adaptation

**Figure 5:**
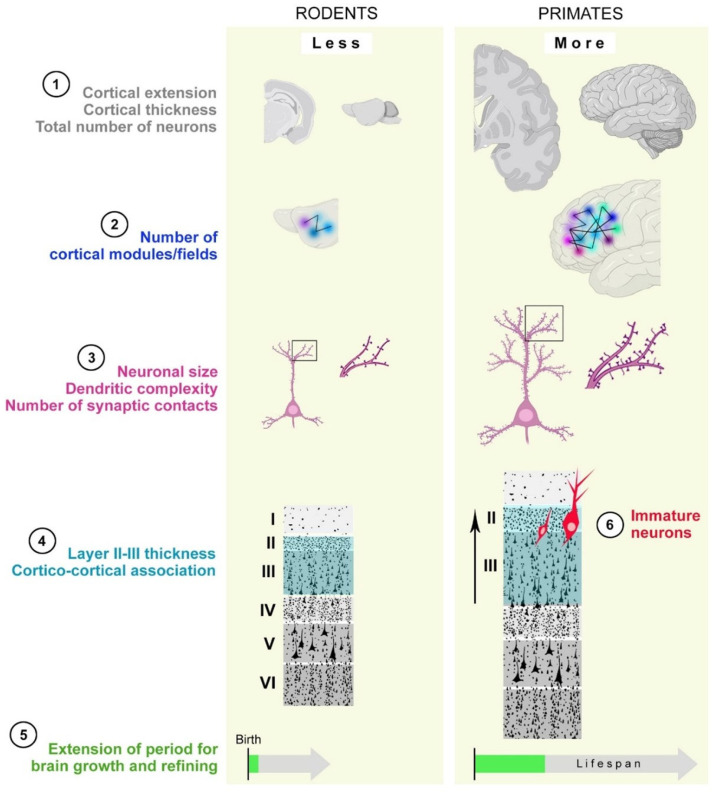
Aspects of cortical neuroanatomy that differ between primate brains and rodents. Points 1 to 5: schematic representation based on data from [151,154,155]. Point 6: the increase in cortical immature neurons in layer II of large-brained, gyrencephalic species. Created with BioRender.com.

Neuronal plasticity, as a dynamic process occurring in a highly interconnected tissue, is undoubtedly a complex matter. Plasticity has adapted over millions of years to a variety of neuroanatomies, which themselves have adapted in diverse species to different ecological niches and functional needs [147]. Such adaptations have sculpted a mix of neuronal plasticity types that coexist in brains characterized by different developmental trajectories, timetables, and neuroanatomical structures. In general, larger brains (see Section 4.1 for a definition of complex brains) appear to rely mostly on synaptic plasticity and immature neurons in cortical and subcortical regions, while smaller and lissencephalic brains retain more proliferative, stem cell-driven neurogenesis in the hippocampus and olfactory bulb [9,125,127]. Qualitative and quantitative studies generally support the trade-off hypothesis [9,35,46,125,127,148,149,150], although these processes do not co-vary linearly [151,152]. Whatever the approach used (comparative neuroanatomy, genetics, epigenetics, transcriptomics, or molecular biology), it is extremely difficult to identify features that might be exclusive to the human brain’s structure and function, setting it apart for its outstanding cognitive abilities [151]. Human cognitive skills, such as language, self-awareness, and other aspects of higher-order thinking, show commonalities in their foundations with the cognition of other animals [151,153]. Thus, identifying the evolutionary changes in the human brain structure that are associated with our specialized cognitive abilities remains an open issue (Figure 5).

### 4.1. Brain Diversity and Primate Evolution

The class of mammals includes nearly 6000 species, displaying remarkable diversity in size, morphology, habitat, and behavior, with their brains similarly reflecting this variability. For example, a mouse weighs about 20 g, with a brain of 0.6 g, whereas certain whales can weigh from 45 to 160 tons, with a brain of 5–10 kg [156,157,158,159]. Despite these differences, we know that all brains, including those of other vertebrates such as fish, reptiles, and birds, share a common structural plan and similar operating mechanisms. In general terms, almost all neuroanatomical structures and cell types found in mice are also found in primates [160]. The use of laboratory rodents as common animal models in the biomedical field is based on these homologies, with the aim of translating research results to humans. However, it is becoming increasingly evident that there are substantial species-specific differences in brains due to the evolutionary process, which can cause challenges in translating basic research in animal models to application in human medicine [40,41,161]. Although large primate brains, particularly those of humans, are often associated with cognitive abilities including attention, memory, language, and planning, these species cannot be meaningfully described as “more evolved” or uniformly “more intelligent.” Even the notion of greater “complexity” is problematic, as high levels of structural or functional interconnectedness can also be found in taxa lacking a true brain, such as insects [162]. No single anatomical feature uniquely accounts for the cognitive capacities of primates. Instead, growing evidence suggests that these abilities arise from the convergence of multiple neural characteristics, rather than from any exclusive or qualitatively novel brain structure (Figure 5). Five such features are outlined below.

(i) A very expanded cerebral cortex that can host a very large number of neurons. Although there are mammals with a larger brain and a more expanded cerebral cortex than ours (cetaceans, elephants, and hippopotamuses), a greater number of neurons are present within the cerebral cortex of large primates. The brain of elephants can reach 5 kg (over three times that of humans) and contains about 250 billion neurons; however, its cerebral cortex (about twice that of humans) contains only 6 billion neurons compared to our 16 billion [163]. Chimpanzees, the primates closest to humans, are estimated to have 7 billion cortical neurons. This alone, however, is not sufficient to explain the cognition of humans and other primates, since some studies suggest that dolphins might have higher numbers of neurons [164].

(ii) Increase in the number of cortical modules. The cerebral cortex can be viewed as a large surface divided into areas differing in cytoarchitecture and function (e.g., sensory area and motor area). Each area is further subdivided into modules, that is, microcircuits of interconnected neurons that form more specialized morpho-functional units. Although the cortices of all mammals have substantially corresponding primary sensory areas, those of larger and gyrencephalic brains contain a much greater number of modules [165,166] and a greater diversification of neuron subtypes [87,167,168]; these features are interpreted as a specialization that may allow more sophisticated processing abilities.

(iii) A very high number of connections. Large brains and expanded cortices generally host larger neurons with longer and more branched dendritic arborizations. In addition to exhibiting more extensive branching, primate dendrites also exhibit a higher number of spines able to form synapses [154,156]. This leads to increased spacing between neurons, with much of the intervening space occupied by dendritic arborizations and the synapses that densely cover them. Therefore, the cerebral cortex of primates and other large-brained species will have not only more neurons in absolute terms and a greater number of microcircuits (see above), but each neuron will also be covered by a greater number of synaptic contacts, thus increasing the number of possible connections [169]. This partly explains why the cerebral cortex in large-brained species is thicker than in mice [170] (see also below). In this regard, the cortical thinning observed in senile dementias, such as Alzheimer’s disease, is largely due to the reduction in synaptic contacts and in the volume of dendritic arborizations, rather than to a decreased number (death) of neurons.

(iv) An increased thickness of the cortical layer II/III. The neocortex of mammals has six layers, five of which are occupied by neurons. The deeper layers host a larger projection of neurons, leaving the cortex to reach other subcortical sites. The superficial layers II and III contain smaller neurons that mainly have an associative function, making projections to different parts located within the cortex itself [171]. These cortico-cortical connections can link the different cortical layers to one another and different areas of the neocortex, with others performing different functions, even between the two hemispheres. These superficial layers are thicker in slow-developing and large-brained primate species, representing a greater percentage of the total cortical thickness compared to what is observed in rodents [155,170,172]. Hence, while projections that perform specific functions (motor and sensory) are rather similar between mammals, certain primate brains, besides having more connections, have proportionally more cortico-cortical associative capacity.

(v) Extension of the period of brain growth and refinement. Brains continue to grow postnatally until young adult ages, allowing refinement of neural circuits based on experience. In primates, particularly humans, this period is highly prolonged, and the process is called bradychrony [151,173,174]. Unlike neoteny, namely the retention of juvenile features in adulthood, bradychrony also involves neural circuits that, despite being substantially assembled, can still be refined. Some common examples are newborn vision, which is still blurred; a child who first learns to walk and then learns to speak; or the brain that, at birth, can distinguish heat, cold, and pain but is not yet able to understand what causes these sensations or what dangers and opportunities they represent. In short, as individuals learn to know the features of the surrounding world, changes in plasticity occur in the brain. While in mice this happens over the course of only 3–4 months, in some primates, it may require years, up to 20–25 in the human species [174]. This prolongation of the learning period and related structural plasticity makes it possible to exploit the greater computational power described in the first four points with an enhanced responsiveness to environmental and social learning through plasticity in its various forms.

### 4.2. Immature Neurons as a Further Element of Specialization

Based on our current knowledge of immature neurons, through their progressive awakening and maturation across an individual lifespan, they might lead to: (i) an increased number of functional neurons [99,105]; (ii) maintenance and/or an increase in the thickness of cortical upper layers (by expanding their dendritic tree; see [9]); (iii) an increased number of new connections [99]; (iv) implementation of the associative layer II [9,109]; (v) implementation of amygdala–cortex cross-talk (with the basolateral complex [53,116]); and, in general, (vi) the maintenance of neotenic features at all ages in regions for cognitive and social tasks. Hence, due to their strategic anatomical location and functional properties, the occurrence and abundance of immature neurons in large-brained species might be considered a sixth element of specialization in large, gyrencephalic brains (represented in Figure 5 for the cerebral cortex).

In long-living species, such as primates, the long-lasting block of maturation in dormant neurons is associated with bradychrony, thus producing a form of neoteny that can protract the recruitment of new neurons across a lifetime, allowing the cortical and amygdalar neural circuits to be modified well beyond youth. The same concepts can be applied to stem cell-driven adult neurogenesis in olfactory and hippocampal regions, but in large-brained mammals, including humans, their influence would be restricted to early periods of life, as is the case for the protracted migration of interneurons.

## 5. Existing Pitfalls and Challenges

### 5.1. Different Levels of Understanding of Immature Neurons in the Piriform Cortex, Neocortex, and Amygdala in Mammals

While canonical neurogenic processes have been extensively studied (more than 14,000 papers are available on PubMed to date), a current limitation in our knowledge concerns immature, dormant neurons. The timing of their awakening, further maturation, and functional integration has only been demonstrated in the piriform cortex of a transgenic mouse [98,99]. Subsequent comparative observations concerning their expansion and prevalence in the cortex and amygdala of large-brained species [53,91,107,109,110], for obvious practical and ethical reasons, are currently not supported by functional experiments, and there is no direct proof that they undergo the same fate in gyrencephalic species and humans. Apart from one study, in which sheep were injected with BrdU during pregnancy, and the prenatal origin of immature neurons in both the paleocortex and neocortex of newborn lambs was subsequently demonstrated [107], no other data are currently available in non-rodent mammals. Similarly, in the amygdala, it has been shown that mouse and sheep immature neurons are born prenatally [93,107], yet in other gyrencephalic species, there are only descriptive studies using different cell markers on postmortem material [53,91,111,112]. In particular, in the amygdala, experiments with DCX-Cre-ERT2/Flox-EGFP transgenic mice have not been replicated, nor have they been in the neocortex, due to the absence of dormant neurons in rodents.

**Figure 6:**
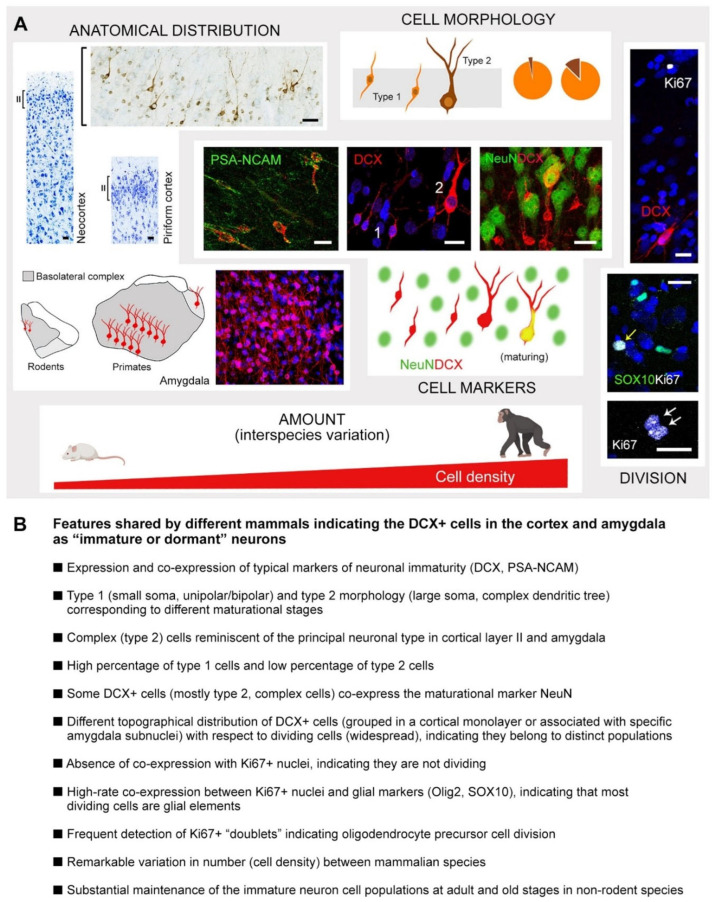
Due to shared cellular markers of immaturity, newborn cells and non-dividing immature neurons can be confused, especially when gyrencephalic species that do not allow invasive functional experiments are considered. To overcome this problem, the convergence of several features shared by immature neurons of widely different mammals can be used to categorize DCX^+^ cells as non-dividing, “dormant” neurons. (**A**) Visual representation of multiple features shared by non-dividing, immature neurons, consisting of morphological, topographical, immunocytochemical, and phenotypic aspects that are consistently observed across widely different mammals. From left to right and top to bottom: They are located in the cortical layer II, forming a strip both in the paleocortex and neocortex, or in the basolateral complex of the amygdala, close to the paralaminar nucleus; they express typical markers of immaturity, e.g., PSA-NCAM, in addition to DCX, and are consistently present in two morphological types: the prevalent (pie charts) small, simple-shaped cells (type 1, orange) and the less prevalent large, highly ramified, pyramidal neurons (type 2, brown; complex cells); type 1 cells are negative for NeuN, while a subpopulation of type 2 cells co-express (yellow) the maturation marker NeuN (green); DCX^+^ cells of both types are never found to co-express the Ki67 antigen, which is occasionally detectable in scattered nuclei within all cortical layers and in the amygdala parenchyma belonging to dividing glial cells, as shown by co-expression of markers for oligodendrocyte progenitors; finally, they show remarkable interspecies variation, from low cell densities in rodents to high cell densities in large-brained, gyrencephalic species. Scale bar: 20 µm. Modified from Ref [110], distributed under the terms of the Creative Commons Attribution License, which permits unrestricted use and redistribution provided that the original author and source are credited. (**B**) Summary of the above-mentioned features. Animal icons created with BioRender.com.

To overcome this problem, in three systematic studies on multiple mammal species [53,109,110], we have defined a series of cellular features that appear to be shared by both cortical and subcortical immature neurons and are well conserved phylogenetically (Figure 6). These features, including highly consistent cellular and molecular characteristics, anatomical distribution, features linked to different degrees of immaturity, and absence of co-expression with cell division markers (Figure 6), match those found in human brains [53,175,176] or transgenic mice [98,99,101,105]. The detection of all these convergent features is at present a way to extend the concept of immature, dormant neurons to gyrencephalic species, especially concerning the neocortex. Besides the use of laboratory rodents to unravel the molecular machinery involved in entering (and exiting) arrested maturation, a future challenge is studying the features of immature neurons in living large-brained, gyrencephalic species, such as their possible modulation in different environmental or pathological conditions.

### 5.2. Immature Neuron Terminology: An Unresolved Issue

Over at least two decades, immature neurons have been gradually recognized as cells in a state of arrested maturation; this was, to some extent, obscured by the strong emphasis on adult neurogenesis (reviewed in [51]). Our current understanding of the cellular and molecular mechanisms underlying the arrest of maturation and the subsequent reactivation of these dormant cells remains incomplete, making this a relatively novel area of research in neuroscience. Accordingly, the small scientific community studying these neurons has not yet reached an agreement on a shared terminology. “Immature neurons” remains a general definition often used to describe other cell types, such as the undifferentiated phase of newborn neurons generated in neurogenic niches, thus generating confusion in the field. As a matter of fact, besides a sharp distinction based on regionalization of most undifferentiated neurons (newborn neurons in neurogenic sites—the hippocampus and SVZ—and immature, dormant neurons in the cortex and amygdala), some brain regions might host a mix of the two types (see Section 5.4).

### 5.3. The Rabbit Exception and Neuroplasticity

**Figure 7:**
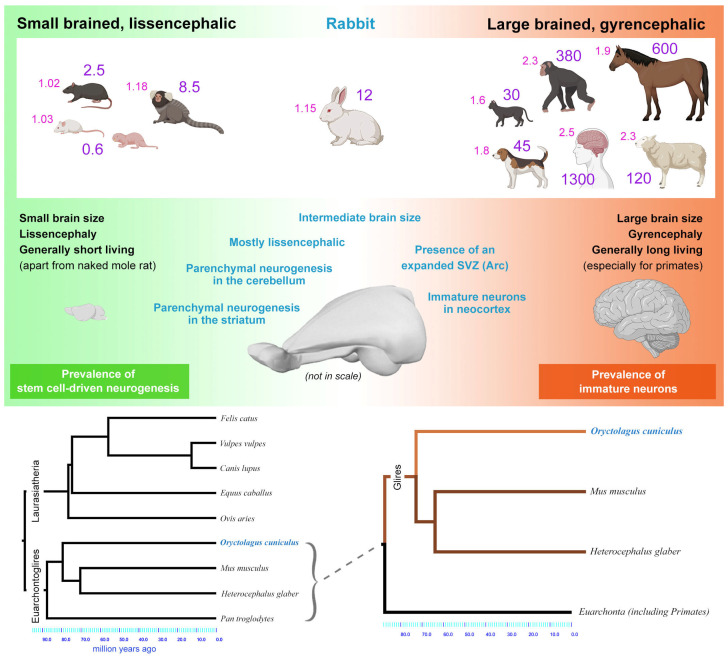
Considering variation in brain size among mammals (large numbers in purple indicate the weight in grams), rabbits occupy an intermediate position, sharing aspects with both rodents and gyrencephalic species (small numbers in pink indicate the gyrification index). Rabbits maintain high rates of neurogenesis, including parenchymal neurogenic events in the striatum and cerebellum, and are, at the same time, the only lissencephalic species hosting layer II immature neurons in the neocortex. Similarly, they have an expanded SVZ (Arc) typical of gyrencephalic species (see Figure 3C). These mixed features might be explained by the placement of rabbits in the phylogenetic tree (bottom), being part of the superorder Euarchontoglires and thus having a common ancestor with both rodents and primates. Animal and brain icons created with BioRender.com.

Our studies on comparative neuroplasticity [52,55,57,95] have revealed an unexpected combination of traits in rabbits. These lagomorphs have a brain size that is somewhat larger than that of many rodents but is lissencephalic [157] (Figure 7, top and middle). Like primates, rabbits possess a well-developed SVZ expansion, hosting migratory chains directed to the cortex (Arc [55,78]; Figure 3C) and cortical immature neurons over their entire neocortical mantle [95,109]. Nonetheless, they also show remarkable neurogenic processes, including parenchymal neurogenesis in the striatum and cerebellum [52,56,57] (Figure 7), and, like rodents, high proliferative potential within the amygdala, mostly linked to oligodendrocyte progenitors [53].

The explanation for these exceptional features in rabbits is currently unclear. These animals are classified within the superorder Euarchontoglires, along with rodents and primates [177,178] (Figure 7, bottom). Being phylogenetically closer to primates and rodents than to the other gyrencephalic species (Laurasiatheria, including Carnivora, Perissodactyla, and Cetartiodactyla) might explain the higher amount of cortical and amygdala immature neurons with respect to rodents, despite being mostly lissencephalic [53,109] (Figure 7).

Overall, rabbits confirm that the evolution of anatomical and functional features can be far from a linear process, thus contributing to the complexity of comparative neuroplasticity. For instance, it has been recently proposed that the cortical folding pattern of primates is strongly determined by brain volume, irrespective of phylogenetic position [179], while the number of immature neurons might also depend on phylogenetic heritage, in addition to brain size [53,109,110] (Figure 7).

### 5.4. Adult Neurogenesis in the Human Hippocampus

**Figure 8:**
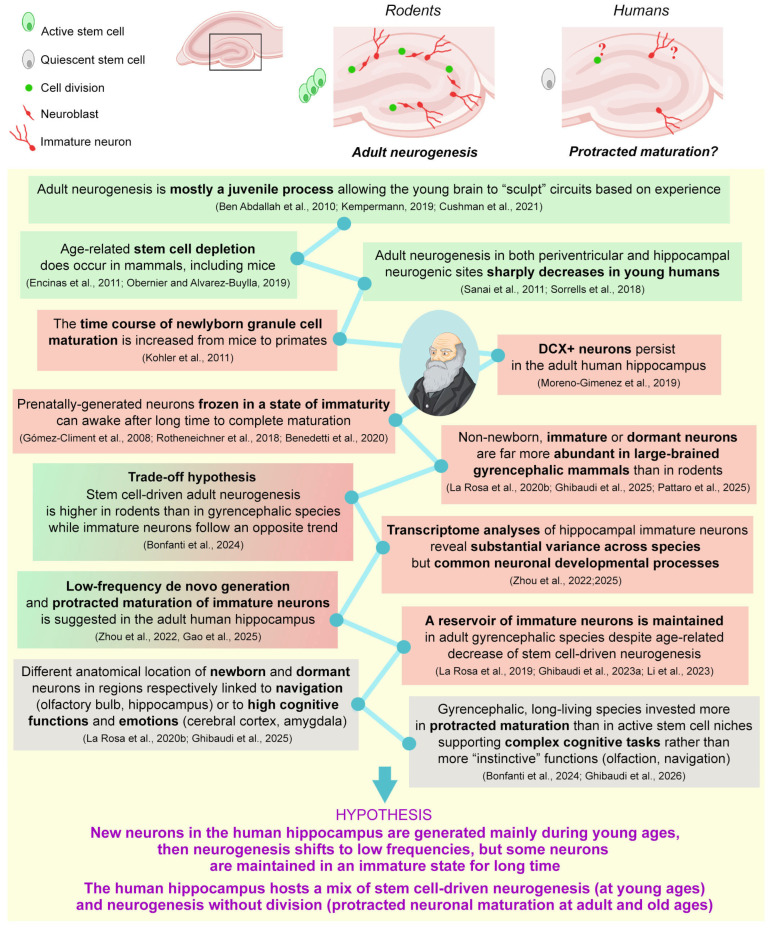
The occurrence and nature of neurogenic processes in the dentate gyrus of the adult human hippocampus have been a matter of discussion for a long time. (**Top**): the occurrence of DCX^+^ neurons in the dentate gyrus seems to be in contrast with the absence of active stem cells hosted in a morphologically defined stem cell niche organization and the consequently very low levels of cell division. (**Bottom**): by linking multiple observations gathered over the years—directly from the human hippocampus and in multispecies studies of widely different brains using histological, immunocytochemical, transcriptomic and phylogenetic analyses—some evolutionary considerations emerge to help in forming an explanation [2,4,9,22,24,25,48,53,94,98,99,104,109,110,125,149,150,175,182,183,184,185,186]. Background color code: green, stem cell-driven neurogenesis; red, immature (dormant) neurons; gray, anatomy and function. The hippocampal section icon created with BioRender.com.

The state of neurogenesis and/or plasticity in the hippocampus of adult humans has been a matter of intense discussion for a long time. Investigators acknowledge the existence of substantial differences between mice and humans, as well as between the young and adult human hippocampi (see Figure 8). Many DCX^+^ neurons are still present in the dentate gyrus of adult humans [48,180] despite a substantial absence of active stem cells hosted in a morphologically defined stem cell niche organization, and consequently, there are very low levels of cell division (likely linked to glial elements). By linking multiple observations, either directly of the human hippocampus or in multispecies studies of widely different brains using histological, immunocytochemical, transcriptomic, and phylogenetic analyses gathered over the years, some tentative conclusions can be drawn (Figure 8 and the references therein). Overall, there appears to be a trend in which large-brained, gyrencephalic, long-living species invest more in the protracted maturation of dormant neurons within brain regions that are involved in higher cognitive functions (cerebral cortex upper layers and basolateral amygdala) than in stem cell-driven neurogenesis linked to olfaction and navigation. In this context, the human hippocampus might be a hybrid region hosting stem cell-driven neurogenesis at young ages, shifting to the protracted maturation of the remaining undifferentiated neurons after stem cell depletion. Accordingly, among the two canonical neurogenic sites, the hippocampus might be more important than olfaction for survival in large-brained, gyrencephalic species due to its important interplay with the cerebral cortex and amygdala [181]. This hypothesis may reconcile multispecies observations and evolutionary trends, yet it remains to be confirmed using novel and reliable approaches for the identification of the last cell divisions required to define the ages of the immature cells in different brain regions and animal species.

## 6. Conclusions and Future Perspectives

This review argues that the growing recognition of interspecies variation in neuroplasticity has increased the conceptual complexity in the field, complicating straightforward translational generalization from rodent models to humans. Historically, observations obtained from laboratory rodents were often assumed to apply to humans, leading to confusion and slowing progress in comparative neuroplasticity research. It has become clear, however, that some cell types, cell markers, and biological processes markedly differ among species due to the complex and non-linear sculpting that took place during evolution [30,125,161]. Phylogenetic differences are evident when considering neurogenic processes due to trade-offs in the types and rates of plasticity [9,125,127].

In conclusion, although recognition of interspecies differences has made the study of brain structural plasticity more challenging, research on comparative neuroplasticity offers a powerful framework for reconstructing the evolutionary landscape underlying human brain organization. Progress will require not only continued technological advances in neuroscience but also more systematic comparative analyses to understand how brain structural plasticity has adapted to diverse mammalian neuroanatomies—knowledge that is essential for meaningful translational research.

## Data Availability

No new data were created or analyzed in this study.
